# Diagnosis of inflammatory myofibroblastic tumor in a pediatric patient initially suspected of tuberculosis

**DOI:** 10.1186/s12887-023-04431-1

**Published:** 2023-11-24

**Authors:** Yiyuan Li, Yang Wen

**Affiliations:** Key Laboratory of Women and Children Diseases, department of pediatrics, West China Second University Hospital, Laboratory of Birth Defects and Related Diseases of Women and Children (Sichuan University), Sichuan University, Ministry of Education, Chengdu, 610041 China

**Keywords:** Inflammatory myofibroblastic Tumor, Tuberculosis, Pediatric

## Abstract

**Background:**

Symptoms of inflammatory myofibroblastic tumor (IMT) are atypical, and histopathological misdiagnosis of IMT is still inevitable. Here we present a pediatric case that an eight-year-old boy with recurrent fever for fifteen months, received anti-tuberculosis therapy for five months and was ultimately confirmed to be IMT.

**Case presentation:**

An eight-year-old boy experienced a recurrent fever for fifteen months, accompanied by cough, vomiting, meteorism, night sweating, and emaciation. Thoracoabdominal computer tomography revealed multiple enlarged lymph nodes in the thorax, abdomen, and axilla, as well as minimal bilateral pleural effusion. Histopathological examinations of the intestines and greater omentum implied fibrous tissue hyperplasia along with eosinophil and lymphocyte infiltration. The patient was initially misdiagnosed with tuberculosis, and symptoms were relieved partially following anti-tuberculosis treatment. However, after four months, the symptoms aggravated again and a subsequent histopathological analysis of a second sample from the greater omentum revealed the presence of IMT. Eventually, after surgical resection of the lesions and chemotherapy, the clinical symptoms in the child gradually alleviated.

**Conclusions:**

The clinical course of IMT is variable, and pediatricians should pay attention to differentiating IMT from tuberculosis.

## Background

The inflammatory myofibroblastic tumor (IMT) is a rare lesion of unclear etiology and variable clinical course. It is characterized by the proliferation of fibroblasts and myofibroblasts intermixed with inflammatory cells. It is a rare tumor classified as a neoplastic disease of intermediate biological potential, given the low risk of recurrence and metastatic potential [1]. Symptoms of IMT are atypical and pathological misdiagnosis and missed diagnoses of this disease are still common. Here we present a case of a pediatric patient suspected of tuberculosis due to recurrent fever, was confirmed to be IMT nearly two years later.

## Case presentation

An eight-year-old male patient was admitted to the hospital for recurrent fever for fifteen months. His personal history, past medical history, and family history were not special. Additionally, there was no known exposure to tuberculosis in the patient’s history.

Fifteen months ago, the child presented with a fever of 40 ℃, accompanied by cough, chest pain, abdominal distension, vomiting, night sweat, and emaciation. The patient was admitted to the local hospital almost once or twice a month due to recurrent fever, and symptoms were temporarily alleviated after antibiotic treatments. Laboratory tests revealed elevated levels of white blood cells (WBC, as high as 18.05 × 10 ^ 9/L), eosinophils (as high as 14.5%), C-reactive protein (CRP, up to 187 mg/L), and erythrocyte sedimentation rate (ESR, up to 115 mm/h). The tuberculosis-related tests, which encompassed sputum cultures for mycobacterium tuberculosis, acid-fast bacilli assessments of sputum smears, purified protein derivative skin test, and gamma interferon release assay, all produced outcomes within the normal range. Thoracoabdominal enhanced computer tomography (CT) scan revealed multiple enlarged lymph nodes in the thorax, abdomen, and axilla, as well as minimal bilateral pleural effusion. Histopathological examination of inguinal lymph nodes displayed reactive hyperplasia, while bone marrow aspiration demonstrated increased eosinophils and a few cells of unknown origin. Laparoscopic examination revealed extensive miliary nodules covering the greater omentum, and histopathological analysis of the mesentery and greater omentum indicated marked fibrous tissue proliferation accompanied by infiltration of numerous eosinophils and lymphocytes. The patient was diagnosed with intraperitoneal tuberculosis; however, the symptoms did not improve after five months of treatment with isoniazid and rifampicin.

Physical examinations were documented. The patient exhibited signs of anemia and severe weight loss. A scar from the Bacillus Calmette-Guerin vaccine was observed on the right upper arm. Palpation revealed enlarged lymph nodes in the bilateral neck, armpit, and groin, with the largest lymph node measuring approximately 1.0 centimeter (cm) × 1.0 cm. The abdomen appeared slightly distended, and a palpable mass measuring about 5 cm × 5 cm was detected at the umbilicus. All other examinations yielded normal results.

**Fig. 1:**
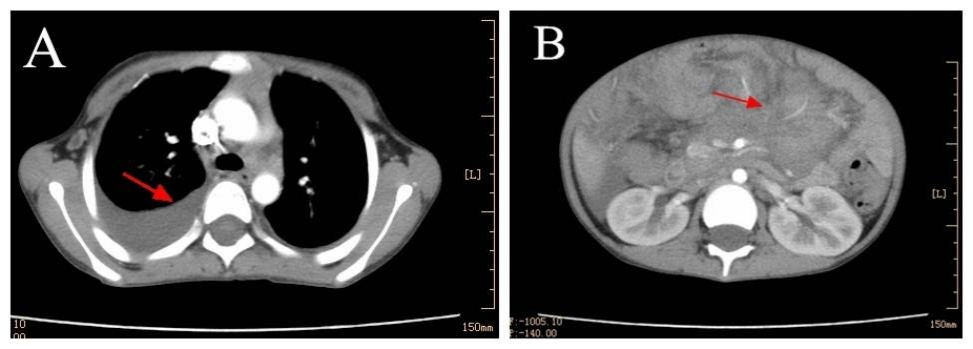
Thoracoabdominal enhanced CT. (**A**) Bilateral pulmonary inflammation, pleural effusion, and pleural thickening, along with enlarged lymph nodes in the mediastinum. (**B**) Thickening and swelling of the intestinal wall in the abdominal cavity

Laboratory tests demonstrated elevated leukocyte count (maximum: 24.5 × 10^9/L), eosinophils (maximum: 31%), platelet count (maximum: 856 × 10^9/L), and C-reactive protein (maximum: 106 mg/L), as well as decreased hemoglobin levels (minimum: 69 g/L). Tuberculosis related tests, including sputum, pleural fluid, and fecal cultures for mycobacterium tuberculosis, acid-fast bacilli examinations of sputum smears, purified protein derivative skin test, and gamma interferon release assay, yielded normal results. Radionuclide bone scans showed no abnormalities. Thoracoabdominal enhanced CT scan revealed bilateral pulmonary inflammation, pleural effusion, and thickening. Numerous enlarged lymph nodes with partial necrosis and significant enhancement were observed in the mediastinum, bilateral hilar regions, axilla, and abdominal cavity. The intestinal wall in the abdominal cavity appeared thickened and swollen, and there was a small amount of fluid in the abdominal cavity and pelvis (Fig. 1).

The patient was diagnosed with fever accompanied by enlarged lymph nodes of unknown origin. Disseminated tuberculosis and lymphoma were considered as potential etiologies. Histopathological analysis of the inguinal lymph nodes indicated lymphoid hyperplasia, while examination of the mesentery and greater omentum revealed significant fibrous tissue proliferation. Additionally, bone marrow aspiration showed no abnormalities, thus ruling out lymphoma as a potential diagnosis. Consequently, tuberculosis infection was considered as the primary diagnosis.

The child was treated with a combination of isoniazid (H), rifampicin (R), pyrazinamide (Z), and ethambutol (E) (HRZE) for anti-tuberculosis therapy, along with meropenem for bacterial infection. After one week, the child’s temperature gradually normalized, and there was improvement in abdominal distension and appetite. Over the next four months of receiving HRZE treatment, the patient remained fever-free for one month, experienced weight gain, showed improvement in abdominal distension and vomiting, and exhibited resolution of lesions on thoracoabdominal CT scans. However, when the regimen was modified to HR, symptoms including fever, abdominal distension, and loss of appetite worsened. Superficial lymph nodes throughout the body became enlarged, and several abdominal masses were detected, with the largest measuring approximately 3 cm × 4 cm. Thoracoabdominal contrast-enhanced magnetic resonance imaging (MRI) revealed pleural and peritoneal effusion, thickening and swelling of the intestinal wall, enhanced thickening with abnormal signal intensity in the peritoneum, greater omentum, and mesentery, as well as multiple enlarged lymph nodes in the abdominal cavity.

During the exploratory laparotomy, numerous miliary nodules were identified attached to the abdominal wall, greater omentum, and intestinal wall. Multiple lesions were surgically excised, with the largest lesion in the greater omentum measuring approximately 7 cm × 5 cm × 2 cm, and the largest intestinal lesion measuring approximately 2.4 cm × 1.6 cm × 0.8 cm. Histopathological features of the abdominal mass, stained with hematoxylin and eosin, revealed the presence of spindle-shaped cells proliferating in patches or nodules, with varying degrees of fibrosis and hyaline degeneration. Mitotic figures were rarely observed. The background showed significant infiltration of inflammatory cells, predominantly eosinophils, along with lymphocytes, plasma cells, and histiocytes. Immunohistochemical staining of the abdominal mass revealed that the spindle cells showed negative expression for epithelial membrane antigen, desmin, S-100 protein, CD34, CD117 and discovered on gastrointestinal stromal tumor-1. The Ki-67/mib-1 proliferation index was 20%, and there was occasional positive staining for smooth muscle actin. Fluorescence in situ hybridization analysis did not detect any anaplastic lymphoma kinase (ALK) gene in the sample. After discussions led by pathologists and hematologists, the patient was ultimately diagnosed with an inflammatory myofibroblastic tumor. Subsequently, the patient was transferred to the department of hematology and oncology for chemotherapy. The chemotherapy regimen consisted of methotrexate (30 mg/m^2^), vinblastine (1.4 mg/m^2^), administered weekly for a total of 10 cycles, in addition to oral administration of prednisone (2 mg/kg, maximum dose of 60 mg). After three weeks, a notable reduction in pleural and peritoneal effusion was observed, and the steroid dosage was progressively reduced over a total treatment duration of six weeks.

The pediatric patient experienced a reduction in abdominal distension and vomiting after surgery, which further alleviated gradually following chemotherapy, with a notable 10 cm decrease in abdominal circumference at the completion of the chemotherapy regimen. One month after chemotherapy, there were no further episodes of fever, and only minimal pleural and peritoneal effusion were observed. Furthermore, laboratory parameters, including WBC, HGB, CRP, and ESR, remained stable within the normal range. Subsequent CT scans conducted in the second and third months post-chemotherapy indicated a reduction in lesions in both the thorax and abdomen. Regrettably, the patient did not seek additional medical care or follow-up after completing therapy.

## Discussion and conclusions

An inflammatory myofibroblastic tumor (IMT) is an intermediate-grade mesenchymal neoplasm that has the potential for recurrence and rare metastasis. IMT is characterized by the presence of myofibroblastic and fibroblastic spindle cells, accompanied by an inflammatory infiltrate consisting of lymphocytes and eosinophils. This type of tumor typically occurs in children and young adults, and the exact etiology and pathogenesis are not fully understood [2, 3]. IMTs can occur in various anatomical locations, with the abdominal cavity, particularly the mesentery, retroperitoneum, and omentum, being the primary sites. Patients with IMT may exhibit atypical symptoms, including fever, weight loss, fatigue, abdominal pain, anemia, thrombocytosis, polyclonal hyperglobulinemia, and an elevated erythrocyte sedimentation rate [3].

The diagnosis of IMT relies on histopathological changes, which can pose challenges. IMT typically presents as a circumscribed nodular mass, although multinodular lesions have also been reported [4]. IMT exhibits a wide range of morphological alterations, including a spindle cell proliferation with a predominantly hyalinized and chronically inflamed background, as well as a highly cellular myofibroblastic proliferation with atypical neoplastic elements. Most tumors are composed of spindle cells, accompanied by fibrosis, hyaline degeneration, calcification, or necrosis. The inflammatory component may be variable, usually consisting of plasma cells, lymphocytes, eosinophils, and neutrophils [4, 5]. Additionally, gene alterations, such as fusion of the ALK gene on chromosome 2p23 with tropomyosin 3, have been detected in approximately 30% of pediatric IMT cases [6]. The pediatric patient in this case presented with atypical symptoms including fever, weight loss, night sweats, abdominal pain, and vomiting, posing challenges in differentiating between tuberculosis and IMT. Given the presence of numerous partially enhanced and necrotic lymph nodes in the thorax and abdomen, along with multiple miliary nodules in the greater omentum during the exploratory laparotomy, and negative findings for lymphoma in radionuclide bone scans, bone marrow aspiration, and histopathology of inguinal lymph node, mesentery and greater omentum, tuberculosis was initially suspected but failed to confirm the diagnosis. Through repeated pathological biopsies of greater omentum and intestines, a definitive diagnosis was eventually established following intense deliberation and analysis. Initial pathological examination revealed spindle cell proliferation and hyalinization in the intestinal wall and omentum, accompanied by infiltration of eosinophils, along with fibroblast or myofibroblast hyperplasia, suggesting inflammatory changes. But the specific causes remained unclear. After discussions and thorough review of the pathological slides, it was further clarified that the predominant inflammatory cells mainly consisted of eosinophils, along with lymphocytes, plasma cells, and histiocytes. There were patchy or nodular fibrosis and hyalinization, consistent with the pathological characteristics of inflammatory myofibroblastic tumor. Taking into consideration the clinical features of the patient, the final diagnosis was made as an inflammatory myofibroblastic tumor. In children, IMT primarily involves the mesentery and greater omentum within abdominal cavity. In cases where there is a clinical suspicion of tuberculosis, particularly abdominal tuberculosis, but insufficient evidence of mycobacterium tuberculosis and unsatisfactory treatment outcomes, early pathological biopsy is crucial. Special attention should be given to pathological characteristic such as myofibroblastic proliferation, chronic inflammatory cell infiltration, and spindle cell proliferation, to differentiate IMT from other conditions. This diagnostic process is complex and requires collaborative efforts among infectious disease specialists, hematologists, and expert pathologists.

Surgery is considered the standard treatment for localized disease, and complete resection offers a high chance of cure. However, the optimal therapy for advanced disease is not precisely defined. Chemotherapy regimens for IMT are disputed. Some researchers in support of chemotherapy suggest that IMT, especially from mesentery and peritoneum, may be potentially malignant by means of local infiltration or distant metastasis [7]. Currently, there was no standardized chemotherapy protocol for IMT. The chemotherapy regimen, commonly utilized in pediatric IMT, consisted of dactinomycin, ifosfamide (or cyclophosphamide), and vincristine, with or without doxorubicin. Additionally, certain cases received treatment involving methotrexate plus vinorelbine, dexamethasone, and ibuprofen [8–10].

## Conclusions

The clinical features of IMT are often atypical, and the pathological diagnosis can be challenging. IMT can be easily mistaken for tuberculosis. Pediatricians should be vigilant in distinguishing between IMT and tuberculosis.

## Data Availability

All data are contained within the article.

## Abbreviations

IMT: Inflammatory fibroblastic tumor
WBC: White blood cells
CRP: C-reactive protein
ESR: Erythrocyte sedimentation rate
CT: Computer tomography
CM: Centimeter
ALK: Anaplastic lymphoma kinase
H: Isoniazid
R: Rifampicin
Z: Pyrazinamide
E: Ethambutol

## References

[1] Bansal A, Goyal S, Goyal A, Jana M (2021). WHO classification of soft tissue tumours 2020: an update and simplified approach for radiologists. Eur J Radiol.

[2] Al Shenawi H, Al-Shaibani SA, Al Saad SK, Al-Sindi F, Al-Sindi K, Al Shenawi N, Naguib Y, Yaghan R (2022). An extremely rare case of malignant jejunal mesenteric inflammatory myofibroblastic Tumor in a 61-year-old male patient: a case report and literature review. Front Med (Lausanne).

[3] Camela F, Gallucci M, di Palmo E, Cazzato S, Lima M, Ricci G, Pession A (2018). Pulmonary inflammatory myofibroblastic Tumor in children: a case report and brief review of literature. Front Pediatr.

[4] Wang Y, Shen L, Yun T, Zhu C, Wang P, Wang S (2021). Clinicopathological features of gastric inflammatory myofibroblastic Tumor: report of five cases. Exp Ther Med.

[5] Takayama Y, Yabuuchi H, Matsuo Y, Soeda H, Okafuji T, Kamitani T, Kinoshita Y, Kubokura N, Sakai S, Oda Y (2008). Computed tomographic and magnetic resonance features of inflammatory myofibroblastic Tumor of the lung in children. Radiat Med.

[6] Mehta B, Mascarenhas L, Zhou S, Wang L, Venkatramani R (2013). Inflammatory myofibroblastic tumors in childhood. Pediatr Hematol Oncol.

[7] Baldi GG, Brahmi M, Lo Vullo S, Cojocaru E, Mir O, Casanova M, Vincenzi B, De Pas TM, Grignani G, Pantaleo MA (2020). The activity of Chemotherapy in Inflammatory Myofibroblastic tumors: a Multicenter, European Retrospective Case Series Analysis. Oncologist.

[8] Favini F, Resti AG, Collini P, Casanova M, Meazza C, Trecate G, Ferrari A (2010). Inflammatory myofibroblastic Tumor of the Conjunctiva: response to Chemotherapy with Low-Dose Methotrexate and Vinorelbine. Pediatr Blood Cancer.

[9] Casanova M, Brennan B, Alaggio R, Kelsey A, Orbach D, van Noesel MM, Corradini N, Minard-Colin V, Zanetti I, Bisogno G (2020). Inflammatory myofibroblastic Tumor: the experience of the European pediatric Soft Tissue Sarcoma Study Group (EpSSG). Eur J Cancer.

[10] Kube S, Vokuhl C, Dantonello T (2018). Inflammatory myofibroblastic tumours – A retrospective analysis of the Cooperative Weichteilsarkom Studien-Gruppe. Pediatr Blood Cancer.

